# Histone 4 lysine 20 tri-methylation: a key epigenetic regulator in chromatin structure and disease

**DOI:** 10.3389/fgene.2023.1243395

**Published:** 2023-08-21

**Authors:** Alejandra Agredo, Andrea L. Kasinski

**Affiliations:** ^1^ Department of Biological Sciences, Purdue University, West Lafayette, IN, United States; ^2^ Purdue Life Sciences Interdisciplinary Program (PULSe), Purdue University, West Lafayette, IN, United States; ^3^ Purdue Institute for Cancer Research, Purdue University, West Lafayette, IN, United States

**Keywords:** H4K20me3, chromatin, heterochromatin, histone, methylation, disease, cancer, homeostasis

## Abstract

Chromatin is a vital and dynamic structure that is carefully regulated to maintain proper cell homeostasis. A great deal of this regulation is dependent on histone proteins which have the ability to be dynamically modified on their tails via various post-translational modifications (PTMs). While multiple histone PTMs are studied and often work in concert to facilitate gene expression, here we focus on the tri-methylation of histone H4 on lysine 20 (H4K20me3) and its function in chromatin structure, cell cycle, DNA repair, and development. The recent studies evaluated in this review have shed light on how H4K20me3 is established and regulated by various interacting partners and how H4K20me3 and the proteins that interact with this PTM are involved in various diseases. Through analyzing the current literature on H4K20me3 function and regulation, we aim to summarize this knowledge and highlights gaps that remain in the field.

## 1 Introduction

In every eukaryotic cell, genetic information is encoded by nearly identical DNA sequences. Tissues and organs achieve their identity through varied gene expression patterns that are critically regulated (Greer and Shi, 2012). Proper regulation of gene expression is partially dependent on packaging DNA into chromatin, a complex of DNA and proteins (Millán-Zambrano et al., 2022). Chromatin is divided into two functional states, initially identified by differential chromosomal staining patterns. Euchromatin corresponds to an open and transcriptionally active conformation of chromatin, while heterochromatin is condensed and transcriptionally inert (Saksouk et al., 2015). The major role for heterochromatin is to protect repetitive regions in the genome from damage and to ensure correct chromosome segregation, thereby preventing genomic instability (Allshire and Madhani, 2018). Heterochromatin can be further classified into two subtypes, facultative heterochromatin which is present in gene-rich regions regulating the expression of genes under specific cellular contexts, and constitutive heterochromatin which is typically found in gene-poor regions, including repetitive sequences such as satellite repeats and transposable elements (Allshire and Madhani, 2018). The fundamental unit of chromatin is the nucleosome, which consist of a histone octamer containing two copies each of histone 2A (H2A), histone 2B (H2B), histone 3 (H3) and histone 4 (H4). Each of these histones can be post translationally modified on their non-globular tail domain, leading to various layers of regulation. These posttranslational modifications (PTMs), that include methylation, phosphorylation, acetylation, ubiquitylation, and others, regulate chromatin structure, accessibility, and hence gene expression independently of changes in the DNA sequence, thus their classification as epigenetic marks (Greer and Shi, 2012).

The PTMs of lysine 20 on histone 4 (H4K20) are conserved from yeast to human and can be classified into the following four states: un (H4K20)-, mono (H4K20me1)-, di (H4K20me2)- and tri (H4K20me3)-methylated (Jørgensen et al., 2013). The different methylation states of H4K20 are established by distinct histone methyltransferases containing SET domains that were first discovered in *Drosophila* (Grigliatti, 1991; Fodor et al., 2010). The lysine methyltransferase, KMT5A (SET8 or PR-Set7) catalyzes H4K20me1 (Nishioka et al., 2002), while KMT5B (SUV420H1) and KMT5C (SUV420H2) catalyze H4K20me2 and H4K20me3, respectively (Schotta et al., 2004; Schotta et al., 2008). In this review we focus on H4K20me3, as it is of particular interest due to its association with many physiological processes including heterochromatin structure (Schoeftner and Blasco, 2009; Yodai et al., 2023), cell cycle regulation (Shen et al., 2010), DNA damage (Shoaib et al., 2018), development (Schotta et al., 2008), cancer (Fraga et al., 2005), and other cellular processes. We discuss the process involved in establishing H4K20me3 and the role of H4K20me3 in heterochromatin structure, including the mediators and regulators of H4K20me3. We also dissect and analyze the different roles for H4K20me3 in normal cellular homeostasis, and in various diseases.

## 2 Role of H4K20me3 in heterochromatin formation and structure

### 2.1 H4K20me3 formation and regulation of transcription

**FIGURE 1:**
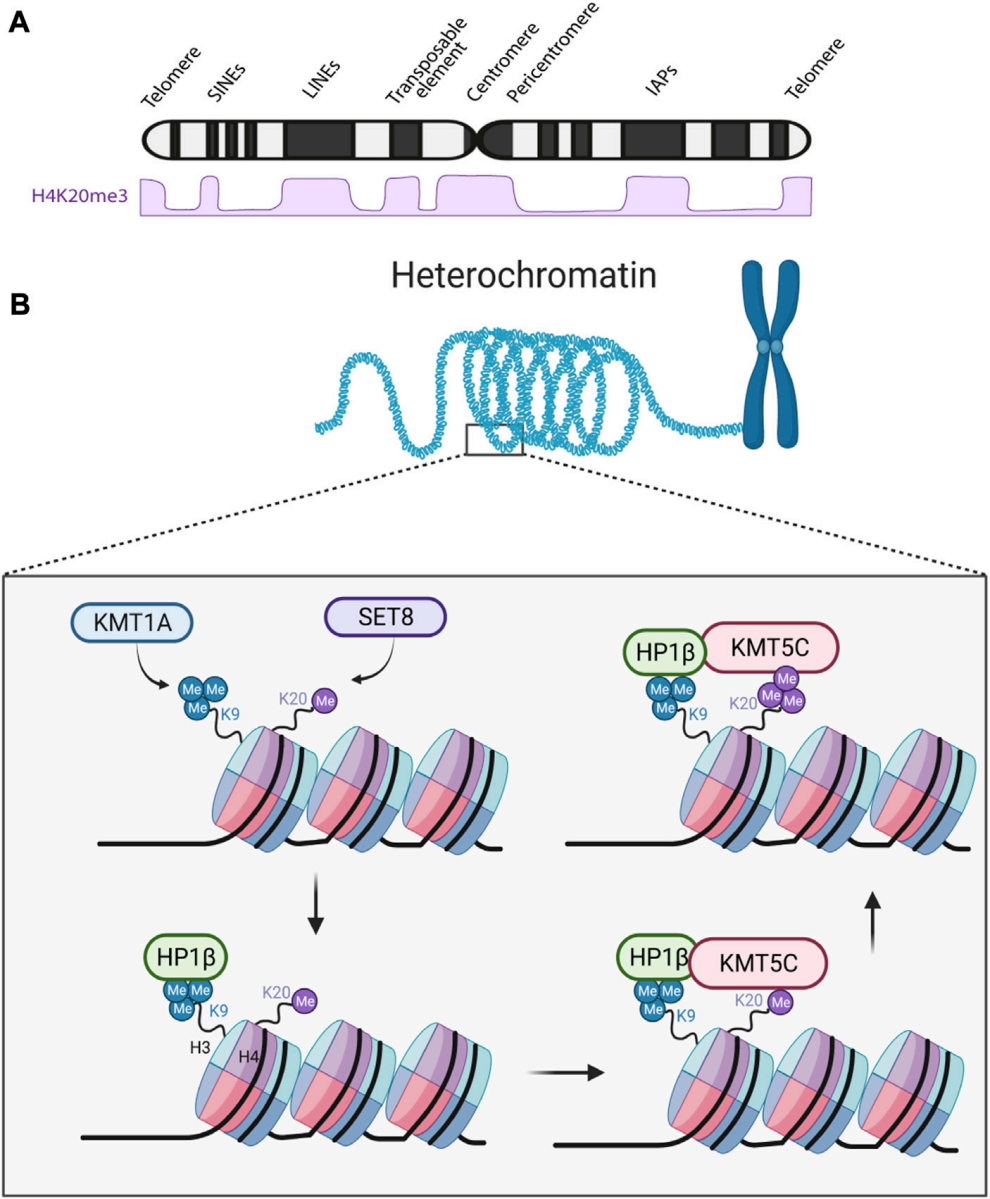
H4K20me3 formation at heterochromatin regions and overall abundance in the chromosome. **(A)** H4K20me3 approximated levels at different regions of the chromosome (Allshire and Madhani, 2018). SINEs, Short interspersed nuclear elements; LINEs, Long interspersed nuclear elements; IAPs, Intracisternal A-particle. **(B)** In constitutive heterochromatic regions of the genome KMT1A methyltransferase induces H3K9 trimethylation, which is then recognized by HP1β (Bosch-Presegué et al., 2017). HP1β then recruits KMT5C to that region, and KMT5C trimethylates H4K20me1 or H4K20me2 (not shown) (Schotta et al., 2008; Hahn et al., 2013). HP1β, Heterochromatin protein 1 β isoform. Created with BioRender.com.

H4K20me3 is abundant at heterochromatin regions that are rich in repetitive sequences, such as satellite repeats and transposable elements, among others (Figure 1A). Heterochromatin is typically associated with reduced transcription (Grewal and Moazed, 2003), and H4K20me3 enrichment at heterochromatin is known to contribute to gene silencing (Saksouk et al., 2015; Cao et al., 2020). H4K20me3 is particularly enriched at repetitive elements, preventing their transcription to maintain genomic stability (Cao et al., 2020). However, recent research suggests that H4K20me3 can also regulate transcription in other genomic regions. For instance, it negatively regulates ribosomal RNA (rRNA) transcription during quiescence and growth arrest (Zhao et al., 2016), as well as the long-non-coding RNA LINC01510 in non-small cell lung cancer (Pal et al., 2022). Repressed gene transcription in heterochromatin is believed to be mediated through chromatin compaction, thereby preventing the binding of DNA factors necessary for transcription (Grewal and Moazed, 2003). Nevertheless, in embryonic stem cells, the association of H4K20me3 with the active transcription methylation mark H3K4me3 suggests a more complex mechanism that requires further investigation (Xu and Kidder, 2018). In summary, the specific localization, and interactions of H4K20me3 in different genomic contexts influence its impact on gene expression.

Furthermore, H4K20me3 methylation is mediated by multiple events, including catalysis by various methyltransferases and their preceding PMTs. These events can be summarized through the following steps. First, unmethylated H4K20 is converted to H4K20 mono-methylation (H4K20me1) by the methyltransferase SET8 or KMT5A (Shoaib et al., 2018). H4K20me1 can also be demethylated and converted to H4K20me0 by the PHD and Jumonji C (JmjC) domain-containing protein PHF8 (Liu et al., 2010). This mono-methylation plays a critical role in cell cycle regulation and in genomic integrity (Shoaib et al., 2018). Following mono-methylation, KMT5B, also known as the *drosophila* homologue SUV420H1 (Suppressor of variegation 4–20 homolog 1), catalyzes the formation of H4K20 di-methylation (H4K20me2) which has an important role in DNA damage response, DNA replication, and in cell cycle regulation (Corvalan and Coller, 2021). H4K20me2 can be converted into its H4K20me1 state by the dosage compensation complex (DCC) subunit DPY-21, a Jumonji demethylase found in both *C. elegans* and mammals (Brejc et al., 2017). Third, KMT5C or SUV420H2 catalyzes H4K20 trimethylation (H4K20me3) (Yang et al., 2008), which is the primary H4K20 PTM involved in heterochromatin silencing (Grigliatti, 1991; Fodor et al., 2010). H4K20, in all its methylation states (me1/2/3), has been reported to be demethylated by hHR23A/B, two human homologues of RAD23 in yeast (Cao et al., 2020). Moreover, reports have shown that H4K20me1 can be used as a substrate of KMT5B and KMT5C, directly generating H4K20me2 or H4K20me3 respectively (Schotta et al., 2004; Schotta et al., 2008; Wu et al., 2013). Indeed, due to the sequence and structural similarity of KMT5B and KMT5C in their catalytic domain, KMT5B and KMT5C have overlapping functions (Schotta et al., 2004). Nevertheless, knockout studies indicate that KMT5B loss leads to a 60% reduction of H4K20me2 with no change in H4K20me3, while KMT5C depletion eliminated H4K20me3 without any significant impact on H4K20me2 in primary mouse embryonic fibroblasts (MEFs) (Karachentsev et al., 2005; Schotta et al., 2008).

While less is known about the events preceding H4K20me3 at facultative heterochromatin, at constitutive heterochromatic regions, another histone PTM has been reported to facilitate H4K20me3 formation (Schotta et al., 2008; Hahn et al., 2013). This PTM, H3K9me3, catalyzed by the KMT1A/SUV39H1 methyltransferase, serves as a docking site for Heterochromatin Protein 1 (HP1) (Schotta et al., 2008; Hahn et al., 2013). HP1 binds to H3K9me3 though its chromodomain which then leads to the recruitment of KMT5C. Once recruited, KMT5C catalyzes H4K20me3 (Figure 1B) (Schotta et al., 2008; Hahn et al., 2013). Previously, it was believed that KMT5C interacted with all HP1 isoforms (HP1α, HP1β, and HP1γ) and that each HP1 isoform interacted with different regions of the KMT5C C-terminus (Vermeulen et al., 2010; Hahn et al., 2013). However, a recent report identified a far more intricate mechanism. While both HP1α and HP1β are enriched at pericentric heterochromatin (PCH) regions, they seem to have opposing roles in chromatin compaction (Bosch-Presegué et al., 2017). Loss of HP1β leads to global decompaction of chromatin through its direct functional link with H4K20me3 and KMT5C (Bosch-Presegué et al., 2017). On the other hand, loss HP1α leads to hyper-compaction of chromatin. Furthermore, when both HP1α and HP1β are lost, HP1γ localization at PCH regions is disrupted (Bosch-Presegué et al., 2017). Thus, the interaction between HP1β and KMT5C appears to be key for H4K20me3 formation at constitutive heterochromatin regions.

### 2.2 The role of H4K20me3 in chromatin structure

#### 2.2.1 The role of H4K20me3 in telomeric heterochromatin

**FIGURE 2:**
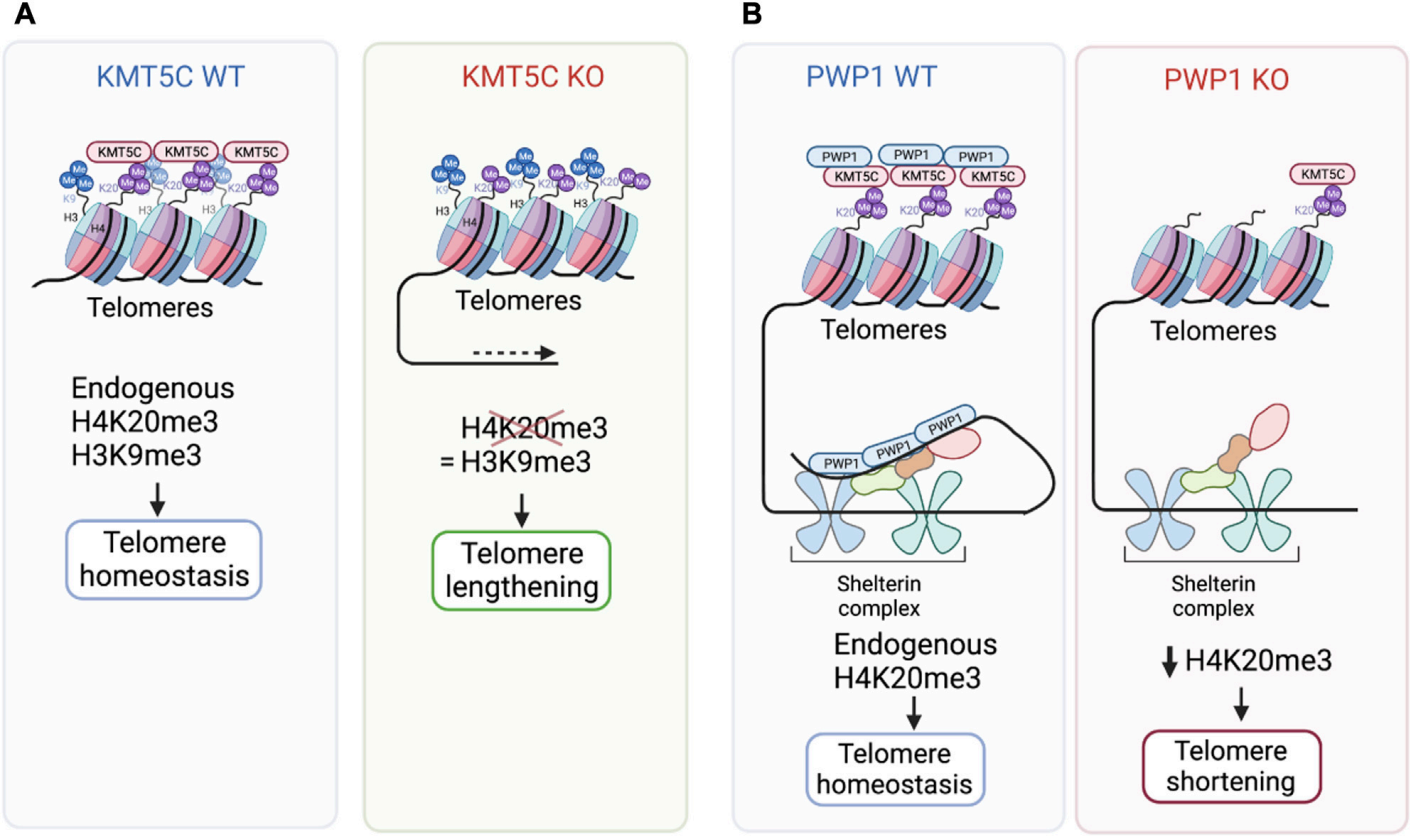
The effect of KMT5C alterations on telomere length. **(A)** KMT5C deficiency reduces H4K20me3 levels at telomeres and subtelomeres, thereby increasing telomeric lenght (Benetti et al., 2007a). **(B)** Loss of PWP1, a protein involved in shelterin complex stabilization reduces H4K20me3 at telomeres and decreases telomere lenght (Yu et al., 2019). PWP1, Periodic tryptophan protein 1. Created with BioRender.com.

Since H4K20me3 is enriched at telomeric regions leading to their compaction and stabilization, its loss has significant implications on telomeric structure and lenght (Schoeftner and Blasco, 2009). Cells deficient for KMT5C methyltransferase, or for both KMT5B/C, have reduced levels of H4K20me3 in both telomeres and subtelomeric regions (Benetti et al., 2007a). This loss is associated with lengthening of telomeres and subtelomeric regions (Benetti et al., 2007a) (Figure 2A). Loss also results in increased sister chromatid recombination globally and at telomeric regions (Benetti et al., 2007a). These changes are specific to loss of H4K20me2/3 as H3K9me3 levels were not affected, lending further support that H3K9me3 is not dependent on H4K20me2/3 (Benetti et al., 2007a). And, while telomere length was altered, there was no evidence of defective telomere capping (Benetti et al., 2007a). A similar telomer lengthening defect was observed following depletion of Retinoblastoma1 (RB1), which is known to interact with KMT5C (Gonzalo et al., 2005). In this work, loss of RB1 led to reduced H4K20me3 and subsequent telomere lengthening in primary mouse embryonic fibroblasts (MEFs) (Gonzalo et al., 2005). However, whether the increase in telomere length when H4K20me3 is reduced is due to more accessibility to telomerase, or if it is through an alternative lengthening of telomeres mechanism (ALT), which relies on the recombination between telomeric sequences to maintain telomeric length was not determined (Benetti et al., 2007a). In contrast to these findings, PWP1, a chromatin binding protein important for controlling growth downstream of mTOR (Liu et al., 2018), has been reported to regulate H4K20me3 levels through binding to and stabilizing the shelterin complex in mouse embryonic stem cells, leading to telomere shortening (Yu et al., 2019). Reduced expression of PWP1 correlated with reduced levels of H4K20me3 at specific telomeric and subtelomeric regions (Yu et al., 2019). PWP1 depletion was also shown to induce telomere shortening and therefore increased DNA damage in telomeric regions (Yu et al., 2019) (Figure 2B). Additionally, restoration of telomere length in PWP1 depleted cells was only achieved by overexpressing PWP1 along with KMT5C (Yu et al., 2019). In support of this association, PWP1 was found to interact with KMT5C along with the shelterin complex, providing regulation of chromatin length (Yu et al., 2019). In addition to proteins such as RB1 and PWP1 that are directly involved in growth, major epigenetic modifiers are also correlated with H4K20me3 levels. In *Drosophila*, loss of the DNA methyltransferase DNMT2, has been correlated with loss of H4K20me3 at retrotransposons and subtelomeric repeats (Phalke et al., 2009). An additional study reported that in telomerase-deficient mice with short telomeres, H3K9me3 and H4K20me3 levels were reduced in telomeric and subtelomeric chromatin, accompanied by increase acetylation of H3 and H4 at these regions (Benetti et al., 2007b). Hence, loss of telomeric repeats appears to lead to loss of heterochromatin features, including H4K20me3. Whether the loss of H4K20me3 in telomeric regions leads to telomere lengthening or shortening is debated and seems to depend on the context. Loss of KMT5C leads to increased telomere length, but loss of PWP1, a protein involved in shelterin complex stabilization, reduces H4K20me3 and decreases telomere length. Contradicting reports, expose the complexity of telomere length regulation which could also be due to differences in experimental design and model systems.

#### 2.2.2 A role for H4K20me3 in constitutive heterochromatin and chromocenter structure

The H4K20me3 mark is also highly enriched in constitutive heterochromatin, including pericentric regions, and thus alterations in H4K20me3 directly impact pericentric chromatin structure and chromocenter structure. Similar to telomeric regions, pericentric regions are also gene-poor and require proper silencing to maintain cell homeostasis. Silencing is reported to be through both H3K9me3 and H4K20me3 (Schotta et al., 2004; Saksouk et al., 2015). Indeed, there is major dysregulation of the 3D chromatin landscape in embryonic stem cells (ES) when KMT5C is lost. This loss results in A/B compartment switching, perturbed chromatin insulation, and altered chromatin interactions of pericentric heterochromatin, indicative of localized decondensation (Kurup et al., 2020). While these findings suggest that KMT5C plays a crucial role in maintaining proper chromatin organization in embryonic stem cells, appropriate chromatin structure in pericentric heterochromatin is also critical during mitosis. Because the pericentric regions plays a crucial role in facilitating sister-chromatin cohesion by recruiting cohesin complexes, any dysregulation in chromatin organization can potentially disrupt this process and result in abnormal cell division. For example, in mouse embryonic stem cells, KMT5C interacts with several cohesin subunits, enabling cohesin binding to pericentric heterochromatin which is essential for correct chromosome segregation (Hahn et al., 2013; Saksouk et al., 2015). KMT5C seems to be involved in the initial loading and maintenance of cohesin subunits in G0-phase cells (Hahn et al., 2013). KMT5B and KMT5C double knock-out cells exhibit reduced chromatin compaction and altered chromocenter organization in interphase, indicating that KMT5C is essential for nuclear architecture (Hahn et al., 2013). The role of KMT5C in pericentric chromatin structure seems to be dependent on a specific region of the C-terminal domain of the protein, a region where HP1 and cohesin interact (Hahn et al., 2013). Nonetheless, additional studies are needed to determine the function of the SET enzymatic domain of KMT5C and H4K20me3 methylation in cohesin recruitment. Similarly, KMT5C seems to play a role in the proper structure of chromocenters and clustering of pericentric heterochromatin (PCH) (Hagihara et al., 2021). Mislocalization of H4K20me3, KMT5C, and HP1 have been observed in cells when the muscle-specific long non-coding RNA ChRO1, is inhibited, which leads to defects in the spatial fusion of chromocenters (Park et al., 2018).

New technologies have provided further insights into the role of H4K20me3 in overall constitutive chromatin structure (Dupont et al., 2023; Yodai et al., 2023). Dupont C. *et al.* utilized a recent imaging methodology, allowing nanometre-scale compaction of chromatin (nanocompaction) quantification in living cells (Dupont et al., 2023). Their study revealed that depletion of both H4K20me2 and H4K20me3 abrogated nanocompaction in embryonic stem cells (Dupont et al., 2023). Additionally, HP1β, known to be functionally associated with H4K20me3, was also found to be crucial in maintaining heterochromatin nanocompaction (Dupont et al., 2023).

In summary, proper regulation of H4K20me3 in constitutive heterochromatin, including pericentric heterochromatin, is critical for maintaining cell homeostasis and normal cellular processes such as mitosis and stem cell function. Dysregulation of these processes, due to loss of KMT5C can lead to chromatin decondensation, altered interactions, and abnormal cell division.

### 2.3 H4K20me3 mediators and regulation

#### 2.3.1 H4K20me3 modification mediators (writers)

**FIGURE 3:**
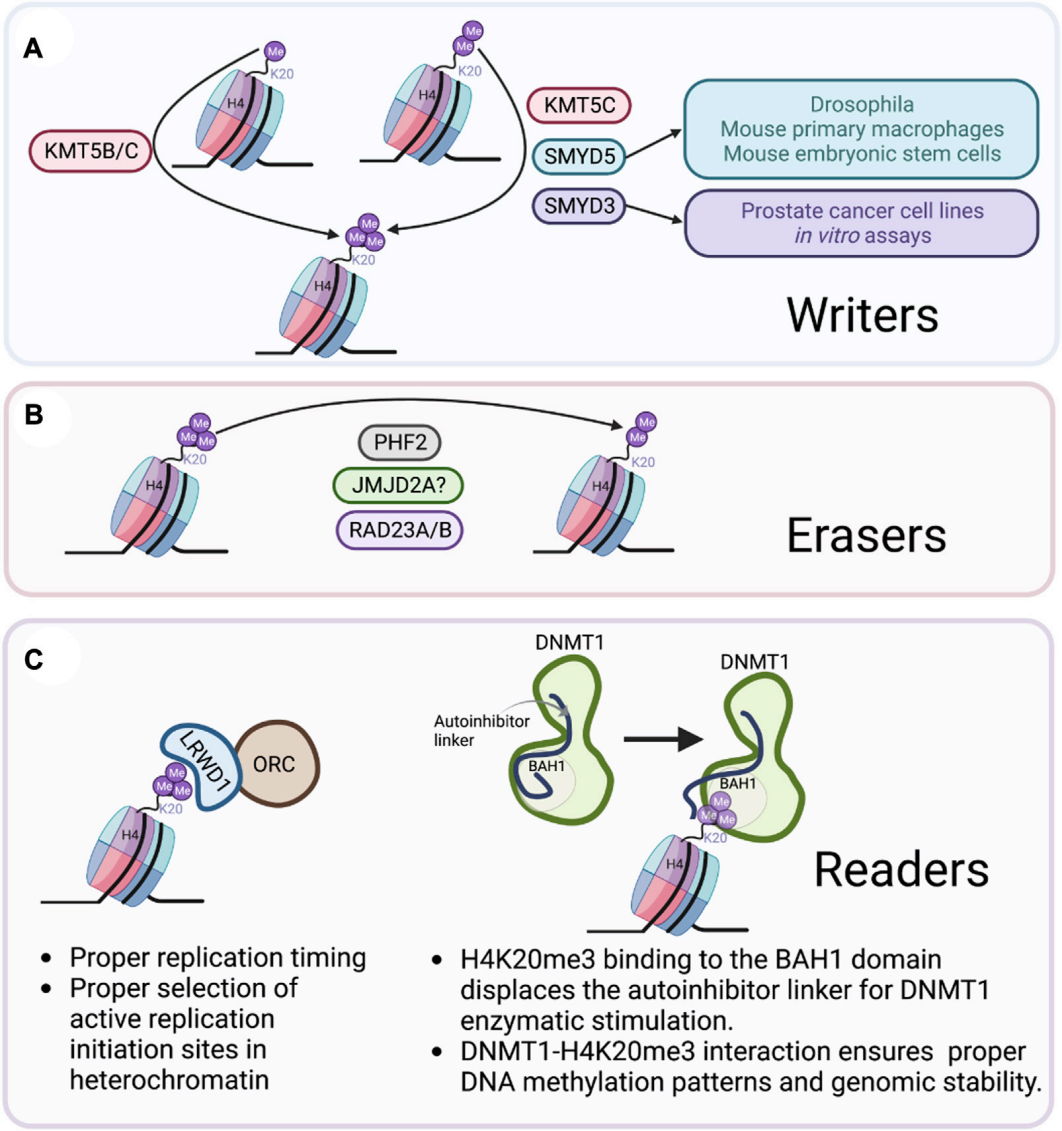
Writers, erasers, and readers of H4K20me3. **(A)** KMT5C (Schotta et al., 2008) is the main writer of H4K20me3. However, the methyltransferases KMT5B (Schotta et al., 2004), SMYD5 (Stender et al., 2012; Kidder et al., 2017a; Kidder et al., 2017b), and SMYD3 (Foreman et al., 2011; Vieira et al., 2015) have been correlated with H42K0me3 formation depending on the cellular context. **(B)** Human RAD23A/B demethylates H4K20me3 in HEK-293T cells (Cao et al., 2020). PHF2 demethylates H4K20me3 at promoters of inflammatory response genes (Stender et al., 2012). JMJD2A recognizes H4K20me3 but demethylase activity has yet to be demonstrated (Lee et al., 2008). **(C)** H4K20me3 readers include an element of the origin of replication complex ORC/LRWD1 (Brustel et al., 2017), and the DNA demethylase DNMT1 (Ren et al., 2021). Created with BioRender.com.

The main H4K20me3 writer is KMT5C (Schotta et al., 2008); although, other methyltransferases have been reported to mediate H4K20me3. SMYD5, a methyltransferase that is a member of the SMYD family of SET and MYND domain-containing proteins, can tri-methylate H4K20 in *drosophila*, mouse primary macrophage cells, and mouse embryonic stem cells (Stender et al., 2012; Kidder et al., 2017a; Kidder et al., 2017b). Likewise, SMYD3, a member of the same family can tri-methylate H3K4 (Chen et al., 2017), H4K5 (van Aller et al., 2012) and H4K20 (Vieira et al., 2015). These SMYD3-mediated methylation events have been observed in prostate cancer cell lines (Vieira et al., 2015) and in *in vitro* assays using histones from HeLa cells (Foreman et al., 2011); however, H4K20me3 was not shown to be a substrate of SMYD3 in other cell line tested, including MEFs, HeLa, and MCF7 cells (van Aller et al., 2012). And, while silencing of SMYD3 was correlated with a decrease in H4K20me3, it appeared to be cell line specific resulting in a need to further validate this correlation with enzymatic assays (Vieira et al., 2015) (Figure 3A).

#### 2.3.2 H4K20me3 erasers

The H4K20me3 mark can be erased by three demethylase enzymes. The first, PHF2, a member of the Jumonji domain family of lysine demethylases, was reported to demethylate H4K20me3 *in vitro* using bacteria purified mononucleosomes, and in macrophages in culture (Stender et al., 2012). PHF2 was found to be involved in removing H4K20me3 at promoters responsible for inflammatory responses (Stender et al., 2012). Whether a similar response would be observed at other regions of the genome has yet to be evaluated (Stender et al., 2012). In agreement with PHF2 acting as an H4K20me3 eraser, its levels were found to be negatively correlated with H4K20me3 levels during mouse embryonic development (Stender et al., 2012). Nonetheless, this could simply be due to reduced expression of KMT5B/C in embryonic chromatin (Eid et al., 2016). The second predicted H4K20me3 eraser is the lysine demethylase JMJD2A, which also recognizes H3K4me3 (Lee et al., 2008). The recognition of H3K4me3 and H4K20me3 is somewhat distinct as the crystal structure of JMJD2A identified a specific mutation (D939R) in JMJD2A that impaired its interaction with H3K4me3 but not with H4K20me3 (Lee et al., 2008). This difference in substrate recognition is useful for technological development and for studying H4K20me3 dynamics; however, the biological and functional significance of JMJD2A in demethylating H4K20me3 remains to be established. The third reported H4K20me3 eraser was identified in a recent screen of ∼2,500 nuclear proteins where a human homologue of the yeast protein RAD23 (hHR23A/B) was identified as an eraser of H4K20me1/2/3 (Cao et al., 2020). Subsequent overexpression of hHR23A or hHR23B in HEK-293T cells reduced the levels of H4K20me1/2/3 (Cao et al., 2020). hHR23A/B demethylation of H4K20me1 activated transcription of coding genes and demethylation of H4K20me3 activated transcription of repetitive elements, further supporting the role of H4K20me3 in repressing repetitive elements in the genome (Cao et al., 2020). Indeed, histone methylation is a very dynamic process mediated by both writers and erasers. In the case of H4K20me3, additional studies of these, and perhaps other proteins will provide important details on the mechanisms that regulates this dynamic process (Figure 3B).

#### 2.3.3 H4K20me3 readers

Readers are proteins that contain a specialized domain that helps them interact with and interpret modifications such as H4K20me3. For example, elements of the origin of replication complex (ORC) were shown to interact with H4K20me3 in a histone marks proteomics study (Vermeulen et al., 2010). More specifically, LRWD1, a leucine-rich and WD40 repeat-containing protein that interacts with an ORC subunit was found to interact with H4K20me3 (Vermeulen et al., 2010). Consistent with this, H4K20me3 is essential to sustain the licensing and activity of a subset of ORCA/LRWD1-associated origins, which allows proper replication timing, and is critical in the selection of active replication initiation sites in heterochromatin regions in mammalians (Brustel et al., 2017). More specifically, H4K20me2/3 serve as enhancers for MCM2-7 helicase loading and replication activation at defined origins (Brustel et al., 2017). The ORC plays a critical role in the initiation of DNA replication and cell cycle progression, highlighting the importance of the H4K20me3 mark in chromatin organization during the cell cycle (Shen et al., 2010).

Additional readers of H4K20me3 are important for maintaining proper DNA methylation, and hence gene silencing. A recent report described a direct link between a DNA methyltransferase and H4K20me3, leading to gene repression (Ren et al., 2021). DNMT1, a DNA methylase important in mitotic division, reads H4K20me3 through binding of its BAH1 domain (Ren et al., 2021). The DNMT1_BAH1_-H4K20me3 interaction triggers a conformational change of DNMT1 into an open conformation (Ren et al., 2021). Disruption of the BAH domain of DNMT1 lead to DNA hypomethylation within an H4K20me3-positive LINE-1 but hypermethylation at genomic regions lacking H4K20me3 (Ren et al., 2021). Hence, the DNMT1_BAH1_-H4K20me3 interaction ensures proper heterochromatin targeting of DNMT1 and DNA methylation at H4K20me3 rich regions such as LINE-1 retrotransposons (Ren et al., 2021). Without question the H4K20me3 function at specific genomic regions intimately depends on the protein or reader interacting with the mark (Figure 3C). It is highly likely that many more readers associating with H4K20me3 across the genome are left to be identified and characterized.

#### 2.3.4 H4K20me3 regulation via KMT5C interacting partners

**FIGURE 4:**
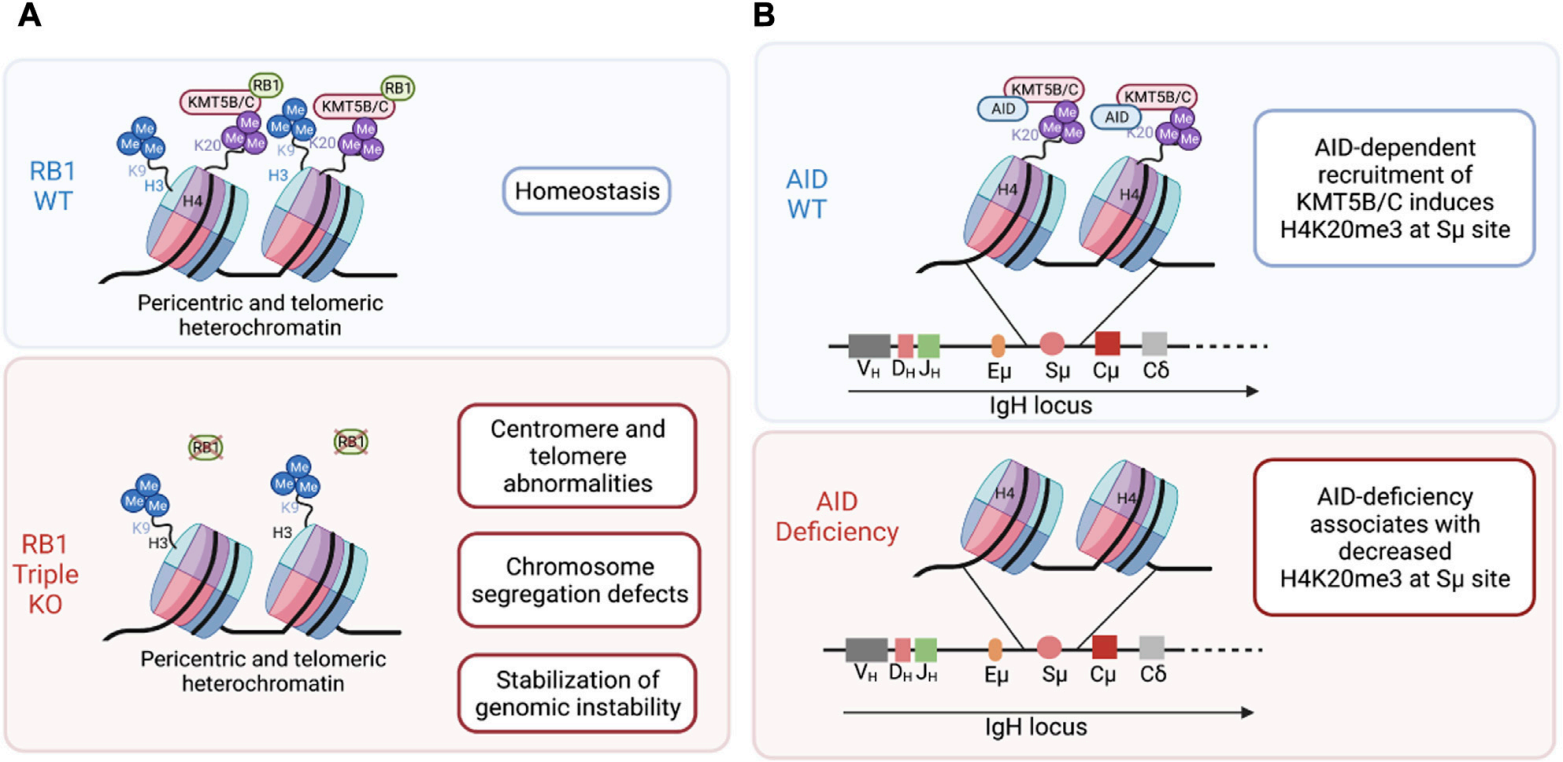
KMT5B/C interacting partners and their effect cellular consequence when they are deleted. **(A)** Deletion of the KMT5B/C interacting partner RB1, leads to genomic instability and chromosome segregation defects (Gonzalo et al., 2005). **(B)** AID deficiency associates with decreased H4K20me3 at Sμ sites (Rodríguez-Cortez et al., 2017). RB1: Retinoblastoma protein 1. AID, Activation-induced cytidine deaminase. Created with BioRender.com.

The process by which KMT5C facilitates H4K20me3 formation is well described. The KMT5C SET domain plays a crucial role in transferring a methyl group from the co-factor S-adenosylmethionine (SAM) to its substrate H4K20me1/2, enabling the tri-methylation at H4K20 (Ferreira de Freitas et al., 2019). However, the mechanism of how H4K20me3 is generated at specific genomic regions is not clearly understood, and identification of KMT5C interacting partners will help elucidate this phenomenon. For example, KMT5C has been reported to interact with members of the tumor suppressor RB1 family, albeit *in vitro* the interaction of RB1 appears to be greater for the H4K20me2 methyltransferase, KMT5B (Gonzalo et al., 2005). Nonetheless, in RB1^–/–^;RBL1^–/–^;RBL2^–/–^ triple knockout mouse embryonic stem cells (MEFs), H4K20me3 levels were reduced in pericentric and telomeric heterochromatin regions, leading to genomic instability and defects in chromosome segregation (Figure 4A) (Gonzalo et al., 2005). Similarly, KMT5C has also been reported to physically interact with the enzyme Activation-Induced cytidine Deaminase (AID) involved in B cells antibody diversification (Rodríguez-Cortez et al., 2017). Interaction of AID and KMT5C led to increased H4K20me3 in specific regions in the genome of human embryonic kidney (293F) cells (Figure 4B) (Rodríguez-Cortez et al., 2017). Furthermore, KMT5C is suggested to interact with Caster Zinc Finger 1 (CASZ1), OIP5 [centromeric and CENPU (both centromeric proteins)] leading to its methylation *in vitro* (Weirich et al., 2016). While the canonical function of KMT5C is to tri-methylate histones, this report suggest that KMT5C can also methylate non-histone proteins in peptide arrays; nevertheless, further research is needed to determine whether these interactions occur *in vivo* or not (Weirich et al., 2016).

#### 2.3.5 H4K20me3 and KMT5C RNA interacting partners

**FIGURE 5:**
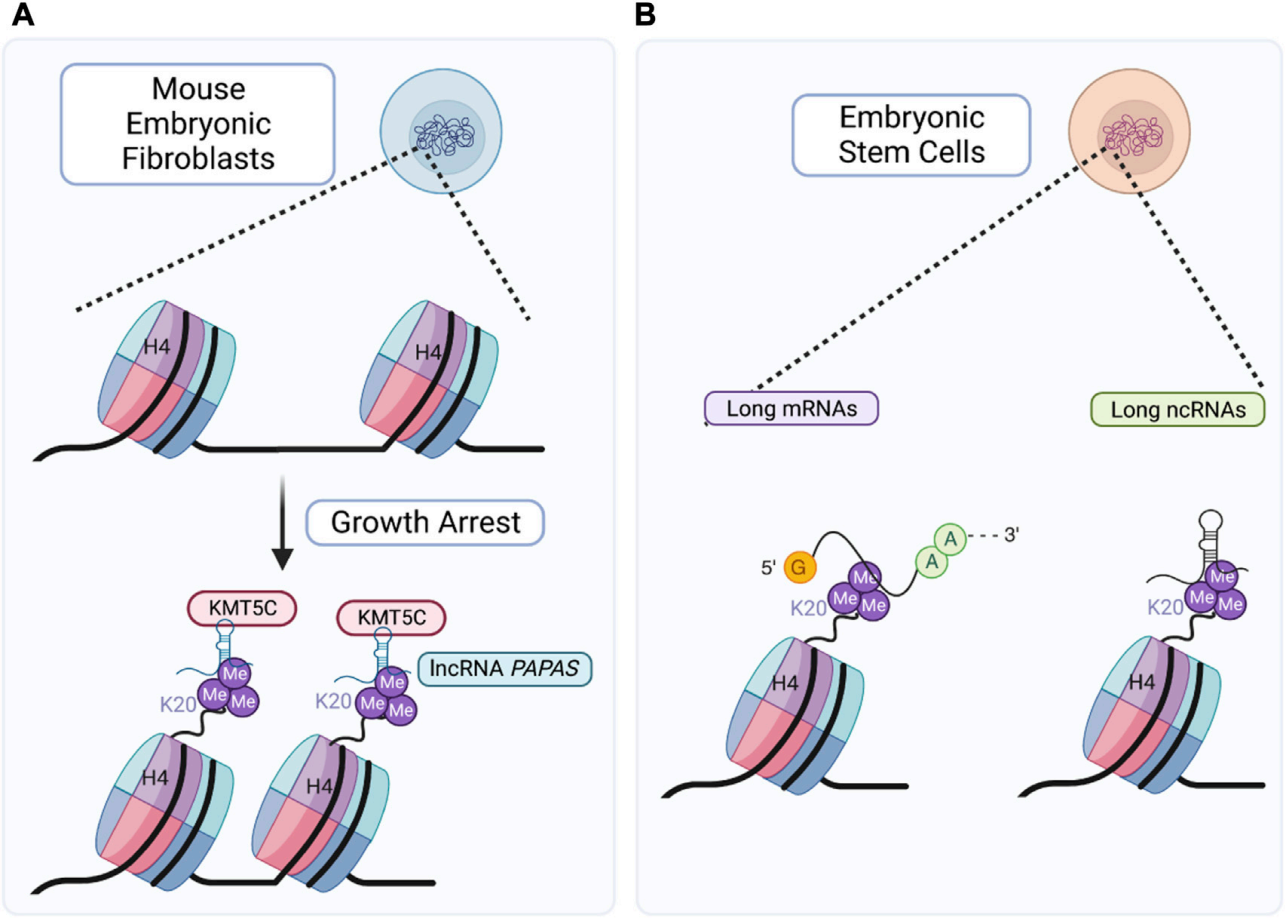
KMT5C and H4K20me3 RNA interacting partners. **(A)** Upon growth arrest, upregulation of *PAPAS* leads to KMT5C mediated H4K20me3 deposition at rDNA regions and IAP elements (Zhao et al., 2016). **(B)** H4K20me3 interacts with long protein-coding transcripts and non-coding RNAs (ncRNAs) involved in DNA repair, cell redox homeostasis, regulation of cell motility/migration, placental development, epithelial cell differentiation (Kurup and Kidder, 2018). Created with BioRender.com.

Furthermore, KMT5C and H4K20me3 have also been demonstrated to interact with both coding and non-coding RNAs, including long non-coding RNAs (lncRNAs). KMT5C was shown to interact with the antisense non-coding RNA (asRNA) PAPAS and regulate ribosomal RNA (rRNA) transcription (Bierhoff et al., 2010). It was first shown that ectopic expression of PAPAS (Promoter and Pre-rRNA Antisense) was able to trigger H4K20me3 increase at chromatin regions containing ribosomal DNA (rDNA), suggesting that antisense RNA could guide KMT5C to rDNA (Bierhoff et al., 2010). Additional studies uncovered a lncRNA-mediated mechanism that facilitates the localization of KMT5C at genomic loci, including rRNA genes and intracisternal A particle (IAP) elements during quiescence or growth arrest (Bierhoff et al., 2014). However, this effect seems to depend on the environmental conditions. Unlike in quiescence and growth arrest, when faced with hypotonic stress, upregulation of *PAPAS* does not increase H4K20me3 in rDNA regions (Zhao et al., 2016). Instead, hypotonicity causes *PAPAS* to increase the interaction between KMT5C and the E3-ubiquitin ligase Nedd4, leading to the degradation of KMT5C (Zhao et al., 2016). Even in the absence of KMT5C, rDNA remains epigenetically silenced due to *PAPAS*' association with a subunit of a nucleosome remodeling complex (Zhao et al., 2016). Clearly lncRNAs guiding of KMT5C to rRNA gene regions depends on the cellular context and hence, further research is needed to discover the regulators of KMT5C and H4K20me3 deposition in regions outside of pericentric and telomeric heterochromatin. Additional reports have also identified RNA association with histone post-translational modifications as a possible mechanism of chromatin and gene expression regulation. Using chromatin-associated RNA immunoprecipitation (CARIP) followed by sequencing, Kurup and Kidder (2018) identified mRNAs and noncoding RNAs that associated with H4K20me3 in embryonic stem cells. It appears that H4K20me3 preferentially interacts with longer protein-coding transcripts and ncRNAs, typically those with a greater number of exons (Kurup and Kidder, 2018). Gene ontology analysis suggests that the transcripts that interact directly with H4K20me3 are involved in RNA processing, DNA repair, cell redox homeostasis, regulation of cell motility/migration, placental development, epithelial cell differentiation, and other processes (Kurup and Kidder, 2018). However, further research is needed to establish whether the interactions for these RNAs with H4K20me3 are involved in heterochromatin formation and/or stabilization (Figure 5).

### 2.4 H4K20me3 in physiology

#### 2.4.1 Cell cycle-dependent tri-methylation of H4K20

Proper balance of histone methylation is necessary to maintain normal biological functions, including proper cell cycle regulation. Indeed, histone methylation at nearly all methylation sites is dynamic with regard to establishment, reversibility, or maintenance across cell division, conferring genomic integrity, stability or reversibility in response to various stimuli (Greer and Shi, 2012). While H4K20me2 is the most abundant methylation state on histone 4 and represents 80% of H4K20 methylation, historic studies determined that H4K20 and H4K20me1 appear to be the most dynamic of the H4K20 methylation states throughout the cell cycle (Pesavento et al., 2008; Greer and Shi, 2012). It was believed that H4K20me1 and H4K20me2 were more closely linked to DNA replication and DNA damage repair than H4K20me3, which was predominantly associated with silenced heterochromatic regions. However, more recent research has elucidated the essential role of H4K20me3 in multiple stages of the cell cycle.

**FIGURE 6:**
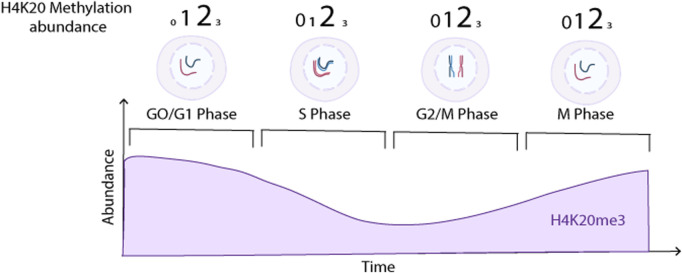
H4K20me3 levels in different cell cycle stages and chromosomal locations. Overall H4K20me0/1/2/3 abundance at different cell cycle stages (indicated by number size). While overall H4K20me3 levels are lower than H4K20me1/2 during the cell cycle, they still oscillate as indicated in the lower portion of the diagram. Created with BioRender.com.

In a homeostatic cell, the levels of H4K20me3 vary depending on the cell cycle stage (Figure 6) and on the levels of H4K20me1/2. In resting cells in G1 or G0 H4K20me3 is high in heterochromatic regions, H4K20me2 is present throughout the genome, and H4K20me1 is restricted to specific genes (Kohi et al., 2022). In early G1, H4K20me1 is reduced as it gets converted to the di- and tri-methylated form (H4K20me2/3) (Kohi et al., 2022). Additionally, due to proteolytic degradation of SET8 during G1, new H4K20me1 modifications are greatly reduced (Kohi et al., 2022). In S phase, during DNA replication when cells are incorporating new histone 4 molecules, very little H4K20me1 is present due to limited SET8 methyltransferase levels (Kohi et al., 2022). However, at the end of S phase, SET8 expression is stabilized resulting in H4K20me1 establishment on most of the new H4 molecules (Kohi et al., 2022). During G2/M and in early mitosis H4K20me1 accumulates and is gradually converted to H4K20me2/3^58^. Following mitosis, in early G1, most of the H4K20me1 is again converted to H4K20me2 and H4K20me3 by the KMT5B/C methyltransferases (Jørgensen et al., 2013).

With the understanding that H4K20 methylation is dynamic during the cell cycle, it is not surprising that deregulation at various stages of the cell cycle can lead to improper replication. For example, in the absence of SET8-driven H4K20 mono-methylation, genome-wide decompaction occurs, leading to excessive loading of the origin of recognition complex (ORC), which can result in DNA damage (Shoaib et al., 2018). Additionally, in early stages of replication, the degradation of SET8 is essential to prevent chromatin compaction caused by further H4K20 methylation. When SET8 degradation is impaired, there are massive defects in cell cycle progression and excessive DNA damage (Beck et al., 2012). This is attributed to elevated H4K20me3, leading to increased ORC recruitment through binding of ORCA/LRWD1 to H4K20me2/3, which then generates aberrant re-replication (Beck et al., 2012). This continued expression of SET8 also leads to increased H4K20me3 in promoters of specific genes, such as histone genes resulting in cell toxicity (Abbas et al., 2010). This results in DNA damage characterized by activation of p53 and G2 checkpoint pathways, leading to increased apoptosis (Abbas et al., 2010). SET8 degradation in early replication is therefore essential for the proper progression of the clockwork-like function of cell cycle, preventing high levels of unregulated H4K20me3 gene repression (Abbas et al., 2010). In the later stages of the cell cycle, such as during mitosis, H4K20me3 regulation is essential for gene repression. In a sequential pattern, proper regulation of H4K20me1 is essential during mitosis because the mono-methylation enables further gene repression mainly generated by H4K20me3 (Karachentsev et al., 2005). While not as intensely studied as some of the other histone PTM, it is becoming increasing clearer that H4K20me1/2/3 levels are very dynamic throughout the cell cycle, and their abundance is essential for proper cell replication and homeostasis.

While the process of methylation is dynamic and critical for cell cycle function, so is demethylation of H4K20. For instance, the PHF8 demethylase acts as a cell cycle regulator by demethylating H4K20me1 allowing for G1-S transition (Liu et al., 2010). During early development in *Xenopus laevis* embryogenesis, the essential explanation for the kinetics of H4K20me1/2/3 in cycling cells involves nonspecific passive demethylation resulting from cell division dilution, rather than active demethylation (Liu et al., 2010). The latter effect also suggests that overall cell-cycle mediated H4K20me dilution through DNA replication is essential for shaping the epigenetic landscape (Schuh et al., 2020).

#### 2.4.2 A role for H4K20me3 in DNA damage

The involvement of H4K20 methylation in the DNA-damage response has been established, and recent reports have started unraveling its underlying molecular mechanism. For example, the known DNA-damage response protein 53BP1 is reported to be recruited to double-strand breaks (DSBs) by direct recognition of H4K20me2, leading to amplification of γ-H2AX signaling (Botuyan et al., 2006; Hsiao and Mizzen, 2013). Although H4K20me1 is also required for 53BP1 binding to DSBs, it is not sufficient and further methylation by KMT5B/C is required for proper 53BP1 nucleation (Tuzon et al., 2014). Recent studies have tried to elucidate the role of KMT5B/C in the DNA repair response by inhibiting the enzymatic activities of these two methyltransferases (Bromberg et al., 2017). Using the KMT5B/C inhibitor A-196, ionizing radiation-induced 53BP1 foci formation was inhibited (Bromberg et al., 2017). Mechanistically, KMT5B/C chemical inhibition repressed non-homologous end joining (NHEJ)-mediated repair but had no effect on homologous-directed repair (HDR)-mediated repair (Bromberg et al., 2017). Henceforth, enzymatic activities of SET8 and both KMT5B/C appear to be essential for 53BP1 recruitment and DSBs DNA repair (Yang et al., 2008; Tuzon et al., 2014).

While H4K20me3 regulation is crucial for the DNA damage checkpoint, it also plays a critical role in regulating subsequent steps in the cell cycle, including the G0 phase. In the presence of various stimuli, cells can exit G1 and enter into G0. Entry into G0 occurs when cells are aging, are under stress, or are inflicted with DNA damage (Di Micco et al., 2021). In this state, otherwise known as senescence, H4K20me3 levels are elevated, specifically at senescence-associated heterochromatin foci (SAHF) (Nelson et al., 2016). Although increased KMT5C expression upregulates H4K20me3, this does not accelerate senescence in normal human cells, but instead reinforces senescence and slows tumor progression in oncogene-expressing cells (Nelson et al., 2016). It is thought that elevated H4K20me3 in senescent cells and aged tissues act as a barrier to cancer through enhancing epigenetic and genetic stability (Nelson et al., 2016). This stability is obtained by suppressing genetic rearrangements that might allow cells to escape from senescence, thereby preventing tumor progression (Nelson et al., 2016). Additionally, cells can also enter G0 when nutrients and growth factors are scarce, this is known as quiescence (Marescal and Cheeseman, 2020). In fact, changes in chromatin compaction and changes in H4K20 methylation are essential for regulating the transition between proliferation and quiescence. In human fibroblasts H4K20me2 and H4K20me3 are increased in quiescent cells (Evertts et al., 2013). Downregulating KMT5C in these cells results in defects exiting the cell cycle and decreased chromatin compaction (Evertts et al., 2013). Hence, H4K20me3 is involved in achieving G0 phase and quiescence (Evertts et al., 2013). However, whether that mechanism is dependent only on chromatin compaction regulation or specific gene repression is still not well understood.

#### 2.4.3 A role for H4K20me3 in the immune response

During immune responses H4K20me3 functions as a molecular checkpoint by regulating class switch recombination. Specifically, H4K20me3 deposited by the methyltransferase SMYD5 works in conjunction with the NCoR corepressor complex to repress expression of toll-like receptor 4 (TLR4) target genes in macrophages (Stender et al., 2012). Upon TLR4 activation, PHF2, a histone lysine demethylase, demethylates H4K20 at these TLR4 promoter regions enabling TLR4 pathway activation (Stender et al., 2012). This puts H4K20me3 methylation/demethylation at the forefront of signal-dependent regulation of inflammatory response genes. However, it is unclear what dictates the specificity that SMYD5 has for its substrate in this context. H4K20me3 levels also appear to interplay with DNA deaminases enzymes to regulate class switch recombination (Rodríguez-Cortez et al., 2017). Activation-induced cytidine deaminase (AID) directly interacts with and recruit KMT5B/C enzymes to Ig switch regions, leading to increase H4K20me3 at these sites, stabilizing class switch recombination function (Rodríguez-Cortez et al., 2017). The latter explains why B cells are defective in class switch recombination in KMT5B/C knock-out mice (Schotta et al., 2008). Similarly, chemical inhibition of KMT5B/C with A-196 can induce class switch recombination, by inhibiting the ability of splenic B cells to switch from IgM to IgG1, IgG3, or IgE (Bromberg et al., 2017). In summary, H4K20me3 contributes to the regulation of immune response genes in specific contexts; however, the mechanisms that dictate KMT5B/C and SMYD5 substrate specificity remain to be established.

#### 2.4.4 A role for H4K20me3 in development

H4K20me3 has been reported to play an essential role in development, stem cell differentiation, and aging. With regard to development in general, the essential role for both KMT5B and KMT5C is clear, as loss of both lead to perinatal death (Schotta et al., 2008). Embryonic lethality is observed when the H4K20me3 demethylases, mHR23A/B, are knocked out, emphasizing the crucial role of H4K20me3 in embryonic development (Ng et al., 2003). The specifics with regard to timing suggest that the dynamics are critical later in development. In mice this is attributed to knowledge that preimplantation embryos lack constitutive heterochromatin markers, including H4K20me3 and HP1α, which appear to be late developmental epigenetic markers (Wongtawan et al., 2011). Hence, H4K20me3 does not mark the onset of differentiation but marks cells in late fetal development when organs and tissues have formed (Wongtawan et al., 2011). In agreement with this, the enzymes that establish H4K20me3, KMT5B/C were mostly absent in mouse embryos before implantation, and ectopic expression of KMT5C led to development arrest (Burton et al., 2020). In agreement, global H4K20me3 sharply decreases after mouse zygote fertilization and starts to increase after implantation (Burton et al., 2020). H4K20me3 function after fertilization likely allows the timely and coordinated progression of replication (Eid et al., 2016). Interestingly, another report has revealed that following fertilization and pre-implantation, mouse embryos experience DNA hypo-methylation (Hatanaka et al., 2015). However, retrotransposons are repressed by histone modifications, specifically H4K20me3, to safeguard genomic integrity during development (Hatanaka et al., 2015). In fact, the histone chaperone chromatin assembly factor 1 (CAF-1) was found to mediate the replacement of H3.3 with H3.1/3.2 at the retrotransposon regions, leading to an increased deposition of multiple histone marks, with a notable emphasis on H4K20me3 and H3K9me3 (Hatanaka et al., 2015). Therefore, it appears that after fertilization and before implantation, the overall levels of H4K20me3 are low except at retrotransposon regions.

In embryonic stem (ES) cells, depletion of SMYD5, a methyltransferase reported to mediate H4K20me3, results in chromosomal aberrations and formation of transformed cells during differentiation (Kidder et al., 2017b). In this context, H4K20me3 was shown to be important for regulating endogenous long terminal repeats (LTR) and long interspaced nuclear elements (LINE)-repetitive DNA elements during differentiation (Kidder et al., 2017b). In fact, SMYD5 was demonstrated to repress lineage-specific genes, and thus, contributed to maintenance of ES cell lineage (Kidder et al., 2017a). Somewhat uniquely to ES cells, H4K20me3 is coupled with activating histone modifications, including H3K4me3 and H3K36me3 (Xu and Kidder, 2018). Association of H4K20me3 with H3K4me3 has been identified in intergenic regions and near transcriptional start sites, whereas H4K20me3/H3K36me3 are located in intergenic regions and within the gene body of actively transcribed genes (Xu and Kidder, 2018). Thus, the role of H4K20me3 in gene expression depends on where in the genome it is located and on its interaction with other methylation marks in ES cells. Whether similar patterns are prominent in other cell types, or during diseases such as cancer that often mirror some of the biology of stem cells, has yet to be reported.

Moreover, in mouse embryonic stem cells, both H3K9me3 and H4K20me3 are regulated by the ten-eleven translocation protein 1 (TET1) (Stolz et al., 2022). TET1 is known to be DNA methylation enzyme, which function includes catalyzing the oxidation of 5-methylcytosine (5 mC) to 5-hydroxymethylcytosine (5hmC), 5-formylcytosine (5 fC), and 5-carboxylcytosine (5 caC) (Stolz et al., 2022). However, a recent report indicated that TET1 has an essential function independent of its catalytic activity (Stolz et al., 2022). TET1 interacts with multiple chromatin modifying complexes, thereby regulating histone modifications, such as the establishment of H3K9me3 and H4K20me3 at endogenous retroviral elements (ERVs) (Stolz et al., 2022). Mice that are TET-deficient do not develop beyond gastrulation, highlighting the importance of H4K20me3 repression of ERVs in mice development (Stolz et al., 2022).

Furthermore, in neuron development, the deposition of H4K20me3 and H3K9me3 has been demonstrated to play a significant role in pericentric heterochromatin (PCH) (Fioriniello et al., 2020). Specifically, during neural differentiation, the methyl-CpG binding protein 2 (MeCP2) physically interacts with major satellite forward RNA (*MajSat-fw*), thereby contributing to the maintenance of PCH by facilitating the deposition of H3K9me3 and H4K20me3 (Fioriniello et al., 2020).

And, while the literature is less established, H4K20me3 changes have also been reported in aging, where increased H4K20me3 levels were observed in the kidneys and livers of aged rats (Sarg et al., 2002).

### 2.5 H4K20me3 in disease

#### 2.5.1 H4K20me3 in cancer

Controlled cell division is crucial to prevent the development of diseases such as cancer. The regulation of induced pluripotent stem cells (iPS), which are often used as a cancer model, can be influenced by various cellular components, including H4K20me3. Abrogation of KMT5B and KMT5C in induced pluripotent stem cells (iPS), leads to loss of H4K20me3 at heterochromatic regions such as telomeres (Marión et al., 2011). The later promotes tumorigenic potential of iPS cells through facilitating telomere elongation during reprograming (Marión et al., 2011). When the function of KMT5B/C is lost, iPS cells *in vitro* are reprogramed, which is characterized by significant epigenetic changes and gene expression patterns similar to that of embryonic stem cells (Marion et al., 2009; Marión et al., 2011). One of the major epigenetic changes observed includes loss of histone and DNA methylation, which leads to a more open chromatin conformation in comparison to differentiated cells. This more “relaxed” chromatin, due to loss of H4K20me3, facilitates access of telomerase to the telomere during iPS cell generation, leading to faithful replication of the chromosome ends (Marion et al., 2009; Marión et al., 2011). And while the telomeres are extended during this process, they are still protected–this is also the case in KMT5B/C double knockout iPS cells (Marion et al., 2009; Marión et al., 2011). These data suggest that H4K20me3 helps to block tumorigenesis in pluripotent stem cells through inhibiting reprogramming and chromatin relaxation (Marión et al., 2011). However, whether loss of H4K20me3 only at telomeric regions is responsible for the malignancy phenotype or if loss at other gene-rich regions is also contributing remains to be elucidated.

Changes in H4K20me3 levels are also aberrantly altered in established tumors when compared to normal tissues. While some studies report elevated levels of the methyltransferase and H4K20me3 in cancer, this is typically the exception as overall loss of H4K20me3 appears to be a more common observation (Fraga et al., 2005). For example, in a panel of tissues and cell lines, H4K20me3 was found to be reduced ∼50% of the time in tumor tissues/cells in comparison to corresponding normal tissues (Fraga et al., 2005). In some tumors, such as in a skin carcinogenesis models, H4K20me3 was progressively lost from early stages to the most malignant stages (Fraga et al., 2005). In lung cancer, loss of H4K20me3 and reduced levels of the *KMT5C* transcript is observed (Van Den Broeck et al., 2008). In addition to reduced H4K20me3 in tumors, loss also appears to be important for generating resistance to various anti-cancer agents. For example, we recently reported on the loss of KMT5C during the process of acquired resistance to EGFR inhibitors in EGFR mutant non-small cell lung cancer cell lines (Pal et al., 2022). Both genetic and chemical inhibition of KMT5C led to resistance, which was partially attributed to loss of H4K20me3 at the locus encoding the long non coding RNA LINC01510, an upstream enhancer of the oncogene MET (Pal et al., 2022). These findings highlight the important role of H4K20me3 as a candidate biomarker for early detection and therapeutic approaches of lung cancer (Van Den Broeck et al., 2008). Besides lung cancer, loss of H4K20me3 is also a marker and molecular contributor to colon, bladder, liver, and breast cancers, and osteocarcoma. In colon cancer H4K20me3 helps with stratification of patient groups–high expression of H4K20me3 is associated with good prognosis in early-stage colon cancer (Benard et al., 2014). In bladder cancer, global H4K20me3 was found to be lower than in normal urothelium tissue (Schneider et al., 2011), while progressive loss of H4K20me3 and decreased expression of KMT5C has been reported in rat liver tumors during hepatocarcinogenesis (Pogribny et al., 2006). In breast cancer cell lines, KMT5C and H4K20me3 levels were found to be low in comparison to normal epithelial breast cell lines (Tryndyak et al., 2006). In this setting, KMT5C was reported to act as a tumor suppressor through H4K20me3 at the tensin-3 locus leading to silencing of the locus and suppression of breast cancer cell invasiveness (Shinchi et al., 2015). Likewise, another report indicated that suppression of KMT5C also results in increased breast cancer cell proliferation (Isin et al., 2020). Similar to the skin carcinogenesis model, expression of KMT5C is lower in breast tumor tissues when compared to adjacent non-cancerous region and higher in early stages of breast cancer when compared to advance stage diseases (Isin et al., 2020). In an additional study conducted in breast cancer cell lines, H4K20me3 gene repression of the tumor suppressor TMS1 limited gene reactivation (Kapoor-Vazirani et al., 2011). In this novel study, H4K20me3-mediated gene silencing involved negative regulation of Pol II promoter escape, thereby enforcing Pol II pausing, leading to gene repression (Kapoor-Vazirani et al., 2011). Finally, in osteosarcoma, reduced levels of KMT5C and H4K20me3 were also observed in tumor tissue and malignant cell lines compared to normal counterparts (Piao et al., 2020). RNA seq analysis after KMT5C knockdown identified pathways involving mitogen-activated protein kinase, p53, and ErbB to be dysregulated; however, whether these genes are directly regulated by H4K20me3 repression remains to be elucidated (Piao et al., 2020).

While the mechanisms that contribute to KMT5C downregulation during tumorigenesis are still incomplete, at least one study points to microRNA involvement. In this body of work miR-29a was shown to directly target the *KMT5C* transcript leading to epithelial to mesenchymal transition (EMT), promoting migration and invasion of breast cancer cells, further supporting a tumor suppressive role for KMT5C (Wu et al., 2019).

Nonetheless, the roles of KMT5C and H4K20me3 in tumorigenesis are clearly context dependent. Expression data from The Cancer Genome Atlas (TCGA) indicates that KMT5C is highly expressed in cancers (Herlihy et al., 2021). And, increased expression of H4K20me3 was reported in esophageal squamous cell carcinoma tumor tissues (Zhou et al., 2019) and in pancreatic cancer, where KMT5C is reported to favor mesenchymal identity, while KMT5C knockdown decreased stemness and increased drug sensitivity (Viotti et al., 2018). In summary, many reports highlight the loss of H4K20me3 in cancer; however, it appears that H4K20me3 selectively represses various targets depending on the biological context, adding another layer of complexity to this epigenetic regulator and its role in cancer progression (Table 1).

**TABLE 1:** Summary table of overall H4K20me3 levels in cancer and other diseases.

Disease	H4K20me3 levels
Skin Cancer Fraga et al. (2005)	↓
Lung Cancer Van Den Broeck et al. (2008)	↓
Colon Cancer Benard et al. (2014)	↓
Bladder Cancer Schneider et al. (2011)	↓
Hepatocarcinogenesis Pogribny et al. (2006)	↓
Breast Cancer Tryndyak et al. (2006), Shinchi et al. (2015), Isin et al. (2020), Kapoor-Vazirani et al. (2011), Wu et al. (2019)	↓
Osteosarcoma Piao et al. (2020)	↓
Pancreatic Cancer Viotti et al. (2018)	↑
Esophageal Squamous Cell Carcinoma Zhou et al. (2019)	↑
Fragile X syndrome Kumari and Usdin (2010)	↑
Obesity Pedrotti et al. (2019)	↑
Obesity/Glucose intolerance Zhao et al. (2020)	↓
Sotos syndrome Berdasco et al. (2009)	↓

#### 2.5.2 H4K20me3 in other diseases

Besides regulating cancer progression, H4K20me3 is also implicated in other diseases. In fragile X syndrome, expansion of the CGGCCG repeat from the Fragile X Messenger Ribonucleoprotein 1 gene leads to gene silencing and to disease development (Kumari and Usdin, 2010). It was found that these silenced alleles are elevated for H4K20me3 (Kumari and Usdin, 2010). However, the mechanism of how H4K20me3 is established at that genomic region, or if it is dependent on KMT5C is not well understood.

In obesity, KMT5B and KMT5C regulate metabolism through downregulating peroxisome proliferator activator receptor gamma (PPAR-γ), which regulates lipid storage and glucose metabolism (Pedrotti et al., 2019). Abrogation of KMT5B/C and therefore H4K20me3, leads to activation of PPAR-γ in brown adipose tissue to increase mitochondria respiration, improve glucose tolerance, and reduce adipose tissue to reduce obesity (Pedrotti et al., 2019). While the data from this work suggest that KMT5B/C proteins may be a therapeutic target for the treatment of obesity, other reports suggest a contradictory effect with regards to KMT5C in obesity, more specifically in adipocytes. Knockout of KMT5C in mice, led to loss of H4K20me3 at the transformation related protein 53 (Trp53) promoter, thereby enhancing Trp53 expression (Zhao et al., 2020). Enhanced Trp53 was found to be responsible for metabolic phenotypes, such as high-fat-diet-induced obesity and glucose intolerance (Zhao et al., 2020). These contradictory findings suggest an ambiguous role of H4K20me3 in obesity, which may depend on whether KMT5C alone or both KMT5B/C are lost.

While there are a modest number of reports highlighting the dysregulation of H4K20me3 in other diseases, more work is needed to uncover whether H4K20me3 is a direct contributor to these diseases or if it is a consequence of the disease. For example, in Sotos syndrome, which is characterized by overgrowth and increased risk of tumorigenesis, H4K20me3 is reduced (Berdasco et al., 2009). This is likely due to loss-of-function mutations of the SET domain-containing protein NSD1 gene (Berdasco et al., 2009). Epigenetic inactivation of NSD1 correlates with diminished methylation of H4K20me3, however whether this effect is dependent on NSD1 SET domain activity has yet to be determined (Berdasco et al., 2009). In sickle cell disease (SCD), activation of the protein arginine methyltransferase PRMT5 induces repressive marks in the γ-globin gene promoter, by assembling a repressor complex that contains KMT5B (Rank et al., 2010). Reactivating γ-globin gene expression and hence inhibiting H4K20me3 in the γ-promoter has been proposed as a potential therapeutic approach for SCD treatment (Rank et al., 2010). Based on these studies it is clear that H4K20me3 and the associated methyltransferases play a major role in normal cellular homeostasis and that dysregulation can lead to deleterious consequence; however, the future depends on gaining further insight into these guardians of the genome and how their misregulation mechanistically contributes to these disease states (Table 1).

## 3 Conclusion and future perspectives

Dysregulation of H4K20me3 is linked to several diseases, including cancer; however, several open questions remain on whether H4K20me3 or KMT5C are good therapeutic targets or not. Due to the potential effects attributed to epigenetic alterations on disease-related genes, oncogenes, and tumor suppressor genes, epigenetic-targeted therapy is becoming a promising strategy for the treatment of cancer and other diseases. First, even though epigenetic modifications are somatically inherited, they can be good therapeutic targets due to their reversibility. Reversibility perhaps makes them more “druggable” than correcting gene sequences (Kelly et al., 2010). Additionally, in diseased cells that are addicted to certain epigenetic abnormalities, using an inhibitor of an epigenetic process can be benificial (Baylin and Ohm, 2006)—for example, when tumor cells become dependent on aberrant gene silencing of oncogenes. As has been the case for other therapeutic modalities, the combination of epigenetic therapies with conventional therapies is worth exploring in preclinical and clinical trials (Ramalingam et al., 2010). Such attempts have already garnered positive results, such as combinations of histone deacetylase inhibitors and DNA repair inhibitors in the treatment of advanced non-small-cell lung cancer (Ramalingam et al., 2010). In addition to therapeutics, epigenetic changes, such as histone methylation, can be used as diagnostics for disease monitoring. For example, studies evaluating H4K20me3 in blood samples determined that H4K20me3 levels can be used as a cancer biomarker (Leszinski et al., 2012). A small clinical study reported that H3K9me3 and H4K20me3 when normalized to nucleosome content could be used as valuable biomarkers to distinguish between cancer patients and healthy patients (Leszinski et al., 2012). In blood samples, H4K20me3 at the centromeric satellites SAT2 was significantly higher in breast cancer and lower in colorectal cancer compared to the respective healthy controls (Leszinski et al., 2012). These blood-based detection methods of H4K20me3 in pericentric heterochromatin-specific circulating nucleosomes represent a potential promising new and non-invasive biomarker for colorectal and breast cancer patients.

While progress has been achieved in utilizing epigenetic therapy to treat various hematological malignancies, further research and clinical trials are necessary to extend the application of these therapeutics to solid tumors and other diseases, primarily due to numerous challenges (Balch et al., 2009; Lin et al., 2009; Cheng et al., 2019). For example, epigenetic events such as the H4K20me3 mark are present ubiquitously across tissues, and selectivity is one of the main challenges. Would it be possible to target inhibitors to particular regions of chromosomes to prevent potential side effects (Kelly et al., 2010)? This is certainly not trivial as targeting inhibitors to various chromosomal regions has yet to be clinically explored. Another challenge in considering H4K20me3 as a therapeutic target is that a clear distinction between changes in H4K20me3 as a causative or merely correlative effect in each disease is essential for the development of successful epigenetic therapies (Kelly et al., 2010). Leveraging KMT5C enzymatic activity as a target, restoring (or inhibiting) such a methyltransferase, has the potential to induce side effects due the complexity of KMT5C regulation and due to its ability to interact with multiple proteins and non-coding RNAs. Hence, simply restoring KMT5C might not lead to the intended therapeutic outcome. In diseases and cancers where inhibition of KMT5C is beneficial, the use of the chemical inhibitor A-196 is a possibility. However, based on other methyltransferases encoded from the genome, the possibility that these other histone-modifying enzymes might compensate for KMT5B/C inhibition and thereby conferring drug resistance needs to be considered (Kelly et al., 2010). It is anticipated that a more thorough understanding on how KMT5C is regulated, how H4K20me3 contributes to gene regulation, discovery of additional readers of H4K20me3, and understanding the role of KMT5C/H4K20me3 in various disease contexts is essential for pushing the therapeutic boundaries (Table 2).

**TABLE 2:** Summary table of the advantages and disadvantages in using epigenetics approaches as therapeutics.

Advantages	Disadvantages
Epigenetic alteration influence expression of disease-related genes, oncogenes and tumor suppressors	Epigenetic selectivity as many methylation marks are ubiquitously present in cells
Epigenetic modification are inherited but are also reversible making them more “druggable”	Epigenetic therapy has to be specific to certain regions of chromosome
Advantages when cancers become dependent on certain epigenetic abnormalities	Difficult distinction between causative and correlative epigenetic alterations
Epigenetic changes such as histone modification can serve as disease biomarkers	Overexpression of epigenetic factors can lead to promiscuity due to lack of proper regulation by other interacting partners
Combination of epigenetics and conventional therapies can be beneficial for cancer treatments	When inhibiting and epigenetic factor, activity compensation by other enzymes should be considered
